# Potential diagnostic value of serum/pleural fluid IL-31 levels for tuberculous pleural effusion

**DOI:** 10.1038/srep20607

**Published:** 2016-02-11

**Authors:** Yan Gao, Qinfang Ou, Jing Wu, Bingyan Zhang, Lei Shen, Shaolong Chen, Xinhua Weng, Ying Zhang, Wenhong Zhang, Lingyun Shao

**Affiliations:** 1 Department of Infectious Diseases, Huashan Hospital, Fudan University, Shanghai 200040, China; 2 Department of Pulmonary Diseases, Wuxi Infectious Diseases Hospital, Wuxi 214005, China; 3 Department of Molecular Microbiology and Immunology, Bloomberg School of Public Health, Johns Hopkins University, Baltimore, MD 21205, USA; 4 Key Laboratory of Medical Molecular Virology, Ministry of Education and Health, Shanghai Medical College, and Institutes of Biomedical Science, Fudan University, Shanghai 200032, China

## Abstract

The aim of this study was to explore the diagnostic value of IL-31 levels in the pleural fluid and plasma to differentially diagnose tuberculous and malignant pleural effusion. We enrolled 91 cases, including tuberculous pleural effusion (TPE, n = 50), malignant pleural effusion (MPE, n = 41), other cases including pneumonia with pleural fluid, pulmonary tuberculosis and healthy people as controls. Whole blood was stimulated with the *M. tuberculosis*–specific antigens and plasma was collected. The multiplex bead-based cytokine immunoassay was employed to measure the levels of various cytokines. IL-31 was found to be the most prominent cytokine (*P* < 0.0001), and with an optimal cut-off value of 67.5 pg/mL, the sensitivity and specificity for the diagnosis of TPE were 86% and 100%, respectively. Furthermore, the tuberculosis-specific IL-31 levels in the plasma of TPE patients were higher than that of MPE patients (*P* = 0.0002). At an optimal cut-off value of 23.9 pg/mL, the sensitivity and specificity for the diagnosis of TPE were 92.9% and 85.7%, respectively. Ultimately, the combination of pleural fluid with the plasma tuberculosis-specific IL-31 levels improved the sensitivity and specificity to 94.0% and 95.1%, respectively. Thus, we identified a novel biomarker for the diagnosis of TPE for clinical application.

Pleural effusion is caused by a variety of diseases, including pulmonary infections, pleural tumour metastasis and tuberculous pleurisy^1^,^2^, and the latter two diseases remain difficult to differentiate in clinical practice. The aetiological diagnosis of pleural effusion is extremely important for the treatment and prognosis of patients. Therefore, highly sensitive and specific biomarkers that are convenient and applicable for the differential diagnosis of pleural effusions are urgently needed.

Interleukin (IL)-31 is a cytokine that was recently identified by Dillon and colleagues^3^. IL-31 has been confirmed to be involved in the pathogenesis of inflammatory diseases such as atopic dermatitis, itching, alopecia, inflammatory bowel disease, airway hypersensitivity and hepatitis B related liver failure^4^,^5^,^6^,^7^,^8^. Tuberculous pleurisy is the most common extrapulmonary tuberculosis clinically^9^. The main mechanism of this disease is a delayed-type hypersensitivity response, which increases the vascular permeability of the pleura and results in the accumulation of proteins and specific lymphocytes in the pleural chamber^10^,^11^,^12^. Thus, we speculated that IL-31 may play a role in the production of pleural effusion. In this study, we measured the levels of *Mycobacterium tuberculosis* (*M.tb*)-specific IL-31 in the pleural fluid and the plasma. The results showed that IL-31 is a highly sensitive and specific biomarker for the diagnosis of tuberculous pleurisy and could readily differentiate it from malignant pleural effusion.

## Results

### Clinical characteristics of enrolled participants

Based on the final diagnosis, 134 enrolled participants were divided into five groups: the tuberculous pleural effusion (TPE) group (n = 50), which consisted of patients with a confirmed or probable diagnosis of tuberculous pleurisy; the malignant pleural effusion (MPE) group (n = 41), which consisted of patients with a diagnosis of malignant diseases; the pneumonia with pleural fluid group (n = 11); the pulmonary tuberculosis (PTB) group (n = 15); and the healthy control (HC) group (n = 17). The TPE group (Table 1) consisted of patients whose pleural fluid was confirmed to be culture-positive for *M.tb* (n = 5) and/or patients who were histologically confirmed by pleural biopsy under thoracoscope to suffer from tuberculosis (n = 17). Patients with probable tuberculous pleurisy (n = 30) were sputum culture-positive for *M.tb* (n = 13) and/or positively responded to anti-tuberculosis medications without other possible causes of pleural effusion (n = 30). The mean age of the enrolled patients was 47.6 (12–84) years old, and 37 out of 50 (74.0%) patients were male.

In the MPE group (Table 1), all cases were histologically confirmed by cytology or pathology, including 35 cases of primary lung cancer, 3 cases of liver cancer with pleural metastasis, 1 case of pleural mesothelioma, 1 case of pleural metastasis from a carcinoma of unknown origin and 1 case of lymphoma. The mean age of the patients was 67.4 (39–91) years old, and 30 out of 41 (73.2%) patients were male.

The pneumonia with pleural fluid group (Table 1) included 10 cases of community-acquired pneumonia (CAP) and 1 case of chronic obstructive pulmonary disease with infection. This group consisted of 9 males and 2 females. The mean age of the patients was 55.8 (29–90) years old.

In the PTB group (Table 1), all patients were free of pleural fluid, and their sputum was confirmed to be culture-positive for *M.tb*. Furthermore, the patients were either treatment naïve or treated with anti-tuberculosis medication for less than 1 week. This group consisted of 10 males and 5 females. The mean age of the patients was 48.7 (16–78) years old.

In the HC group (Table 1), 17 individuals were interferon-γ release assay (IGRA)-negative and exhibited no evidence of active tuberculosis. This group included 7 males and 10 females, and the mean age of the patients was 38.9 (25–69) years old.

All enrolled participants were HIV-negative, had not been diagnosed with cancer, diabetes, autoimmune diseases or other chronic infections (i.e., chronic HBV/HCV infection) and had not received any immune-modulating treatments.

### Cytokine levels in tuberculous and malignant pleural fluid

**Figure 1:**
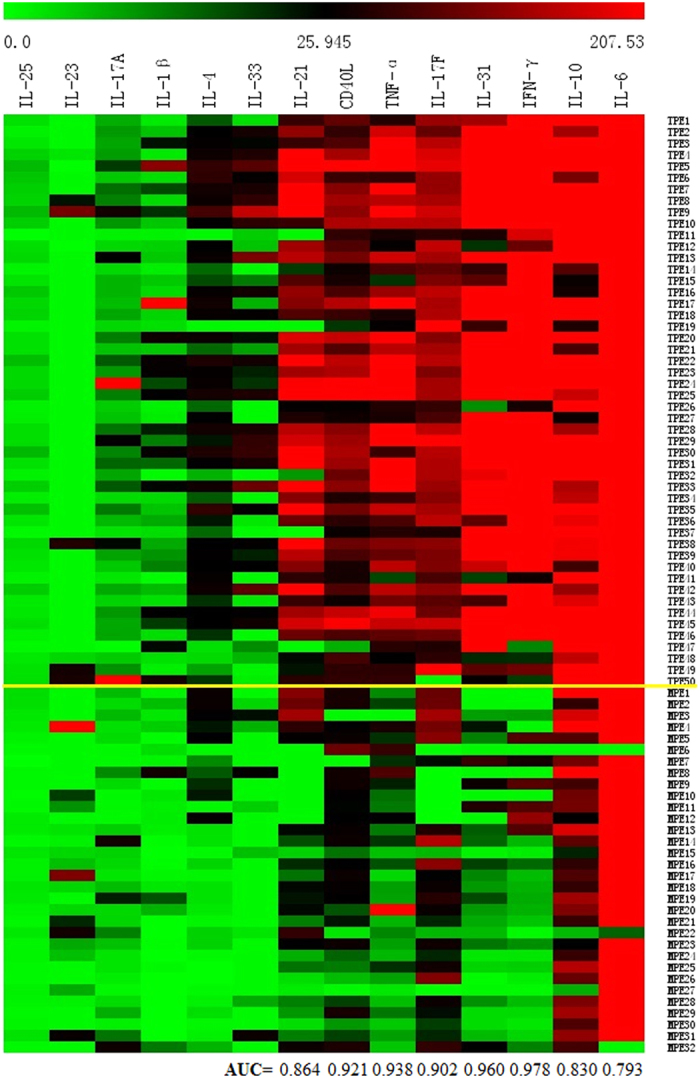
Heat map of cytokine expressions in the tuberculous and malignant pleural fluid. The IL-25, IL-23, IL-17A, IL-1β, IL-4, IL-33, IL-21, CD40L, TNF-α, IL-17F, IL-31, IFN-γ, IL-10, and IL-6 levels in pleural fluid were measured with a multiplexed bead-based cytokine immunoassay. The heat map above the yellow line is the TPE group, and the heat map under the yellow line is the MPE group. TPE: Tuberculous pleural effusion; MPE: Malignant pleural effusion; AUC: area under the curve.

The levels of IL-25, IL-23, IL-17A, IL-1β, IL-4, IL-33, IL-21, CD40L, TNF-α, IL-17F, IL-31, IFN-γ, IL-10, and IL-6 were detected in the pleural fluid. We found that the IL-21, CD40L, TNF-α, IL-17F, IL-31, IFN-γ, IL-10, IL-6 levels in the TPE group were higher than the respective levels in the MPE group (Fig. 1). The receiver operating curve (ROC) assay indicated that the areas under the curve (AUCs) of the above 8 cytokines were 0.864, 0.921, 0.938, 0.902, 0.960, 0.978, 0.830 and 0.793, respectively (Fig. 1).

### IL-31 levels in tuberculous and malignant pleural fluid and the value of these levels in the differential diagnosis of pleural effusion

**Figure 2:**
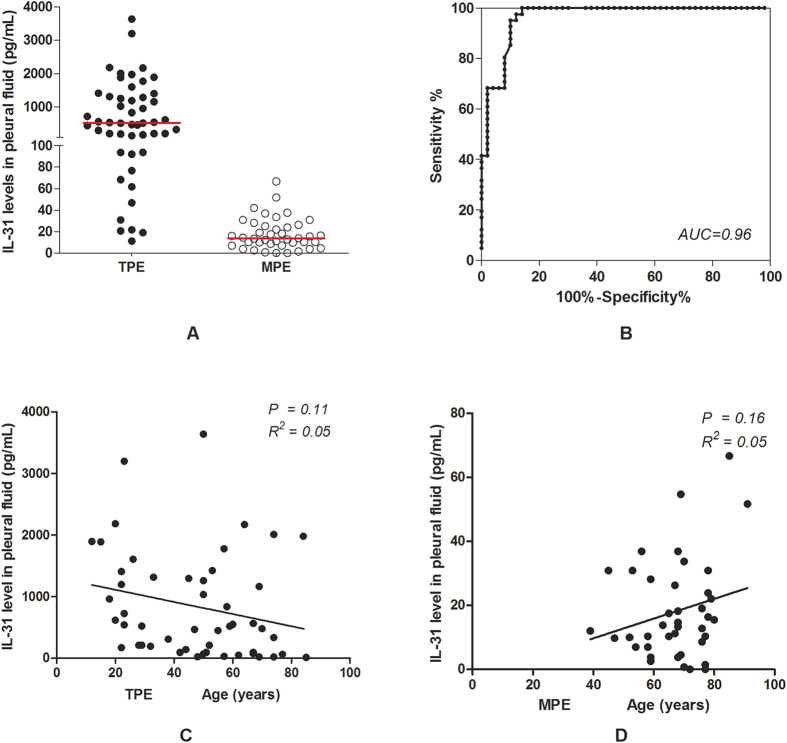
IL-31 levels in the pleural fluid of patients with TPE and MPE. (**A**) The IL-31 levels in the pleural fluid were higher in patients with TPE than in patients with MPE. The short transverse line represents the median of different groups. (**B**) The receiver operating curve (ROC) analysis of IL-31 in the pleural fluid of TPE versus MPE patients. The area under the ROC (AUC) was 0.96. (**C**) Age was not correlated with the IL-31 levels in the TPE group (P = 0.11; R2 = 0.05). (**D**) Age was not correlated with the IL-31 levels in the MPE group (P = 0.17; R2 = 0.06). TPE: Tuberculous pleural effusion; MPE: Malignant pleural effusion.

The median IL-31 levels in the TPE group was 529 4pg/mL (25–75% percentile, 129.5–1339.0 pg/mL), which was dramatically higher than that in the MPE group (13.8 pg/mL; 25–75% percentile, 7.8–25.7 pg/mL) (*P* < 0.0001) (Fig. 2A), and this levels was associated with a high AUC value of 0.96 (Fig. 2B). To elucidate the influence of age on this difference in IL-31 expression, we further analysed the correlation between age and IL-31 levels. However, age was not correlated with the IL-31 levels in neither the TPE group (*P* = 0.11; R^2^ = 0.05; Fig. 2C) nor the MPE group (*P* = 0.17; R^2^ = 0.06; Fig. 2D).

We further determined the optimal IL-31 cut-off value of 67.5 pg/mL in the pleural fluid by ROC curve. With this cut-off value, the sensitivity and specificity for the diagnosis of TPE were 86.0% (95% CI: 73.3–94.2%) and 100% (95% CI: 91.4–100.0%), respectively, whereas the sensitivity and specificity for the diagnosis of MPE were 100.0% (95% CI: 91.4–100.0%) and 86.0% (95% CI: 73.3–94.2%), respectively (Table 2). Thus, the diagnostic accuracy of the IL-31 levels in pleural effusion was 92.3% (84/91).

### The whole blood IL-31 release assay in response to *M.tb*-specific antigens of TPE and MPE

**Figure 3:**
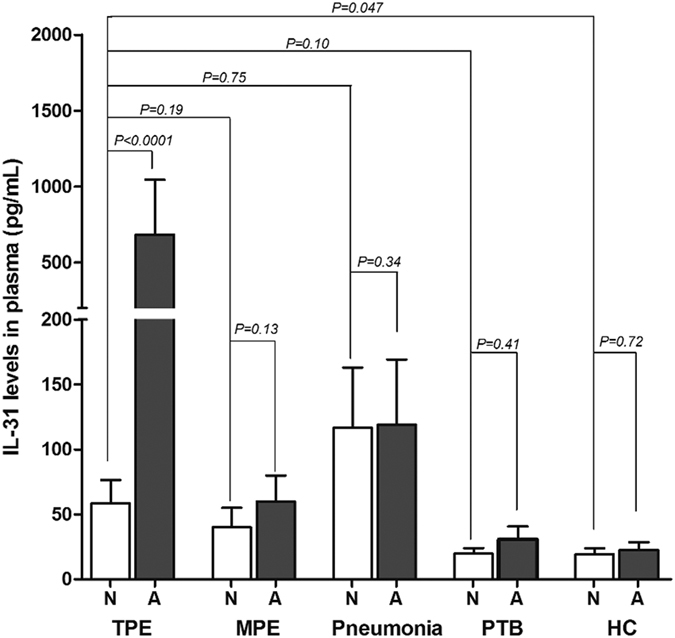
The tuberculosis antigen-stimulated (**A**) and un-stimulated (**N**) plasma IL-31 levels in the TPE, MPE, pneumonia with pleural fluid, PTB and HC groups. The bars represent the SEM. TPE: Tuberculous pleural effusion; MPE: Malignant pleural effusion; PTB: pulmonary tuberculosis patients; HC: health control; SEM: standard error of the mean.

**Figure 4:**
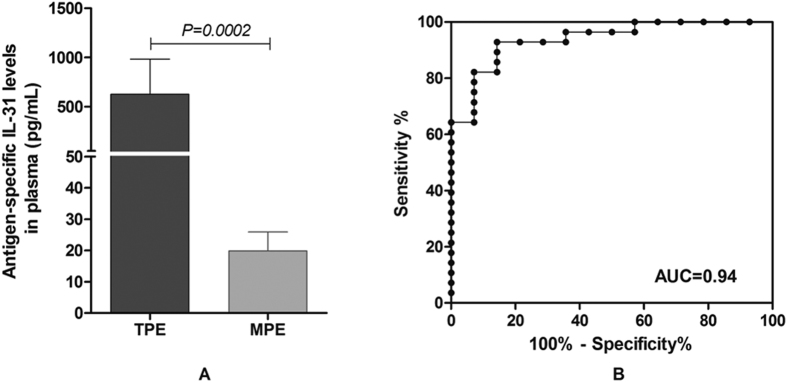
Tuberculosis-specific IL-31 levels in the plasma of patients with TPE and MPE. (**A**) The *M.tb*-specific IL-31 levels in the plasma of patients with TPE were higher than that in patients with MPE. The tuberculosis-specific IL-31 levels in the plasma were determined by subtracting the un-stimulated levels from the antigen-stimulated levels. (**B**) The receiver operating curve (ROC) analysis of the tuberculosis-specific IL-31 levels of TPE vs. MPE patients. The areas under the ROC (AUCs) were 0.94.TPE: Tuberculous pleural effusion; MPE: Malignant pleural effusion.

To further improve the diagnostic power of IL-31 for the differential diagnosis of TPE and MPE, we employed the whole blood IL-31 release assay to compare the antigen-specific IL-31 levels in the two groups. In this study, we recruited patients with pneumonia with pleural fluid, patients with active pulmonary tuberculosis (PTB) and healthy individuals, which served as controls. Without antigen stimulation, the median IL-31 levels in the plasma of the TPE and MPE groups did not differ significantly (24.9 vs. 9.1 pg/mL, *P* = 0.186), and those in TPE, PTB and HC groups were not significantly different (*P* > 0.05) (Fig. 3). Only in the pneumonia with pleural fluid group, the IL-31 levels of the plasma were higher than those in the TPE group (*P* = 0.047), while the IL-31 levels in this group did not increase by antigen stimulation compared to that without stimulation (*P* = 0.34) which could be used for differentiation (Fig. 3). Interestingly, the *M.tb*-specific antigen-stimulated IL-31 levels of the plasma in the TPE group were higher than those in the other four groups. Specifically, the *M.tb*-specific antigen-stimulated levels of IL-31 of the plasma in the TPE group was higher than that in the MPE group, i.e., 200.3 pg/mL (25–75% percentile: 74.7–611.0 pg/mL) and 29.3 pg/mL (25–75% percentile: 23.7–52 7pg/mL), respectively (*P* = 0.002) (Fig. 3). We calculated the antigen-specific IL-31 levels by subtracting the value of the un-stimulated control from the stimulated value, which yielded median values of 176.1 pg/mL (25–75% percentile, 50.6–466.5 pg/mL) and 16.7 pg/mL (25–75% percentile: 6.5–19.1 pg/mL) for TPE and MPE, respectively (*P* = 0.0002) (Fig. 4A). The IL-31 levels did not significantly differ among the pneumonia with pleural fluid, active pulmonary tuberculosis (PTB) and healthy control groups. The ROC analysis of the *M.tb*-specific IL-31 levels in the TPE and MPE two groups yielded an AUC of 0.94 (Fig. 4B) and an optimal cut-off value of 23.9 pg/mL. Using this cut-off value, the sensitivity and specificity for the differential diagnosis of TPE and MPE were of 92.9% (95% CI: 76.5–99.1%) and 85.7% (95% CI: 57.2–98.2%), respectively.

### The combination of IL-31 levels in the pleural fluid with the *M.tb* specific whole blood IL-31 release assay for the diagnosis of tuberculous pleurisy

**Figure 5:**
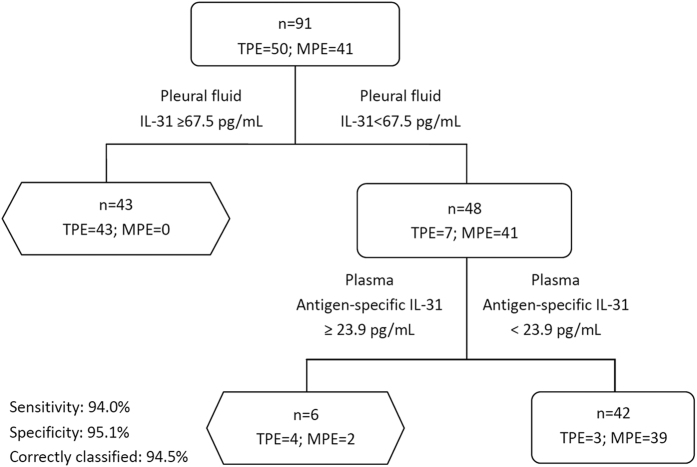
The diagnostic value of the IL-31 levels in the pleural fluid and plasma for tuberculous pleurisy. First, the result of the test was considered positive if the concentration of IL-31 in pleural fluid ≥67.5 pg/mL. Thus, 48 patients were judged to be negative. Second, these 48 patients were considered to be positive if the concentration of antigen-specific IL-31 in the plasma ≥23.9 pg/mL. Thus, 6 cases were judged to be positive. The sensitivity and specificity of the combination of the pleural fluid and plasma IL-31 levels were 94.0% and 95.1%, respectively.TPE: Tuberculous pleural effusion; MPE: Malignant pleural effusion.

Because both the IL-31 levels in the pleural fluid and *M.tb* specific whole blood IL-31 release assay were highly sensitive and specific for the diagnosis of tuberculous pleurisy, we sought to combine these assays to improve the diagnostic power of IL-31 measurement for clinical use. Because the optimal cut-off IL-31 levels in the pleural fluid was 67.5 pg/mL, the test was considered positive for IL-31 levels ≥ 67.5 pg/mL. According to this criterion, 43 and 7 cases in the TPE group (n = 50) were positive and negative, respectively, but all 41 cases in the MPE group were negative. We then used the optimal cut-off value of 23.9 pg/mL for the *M.tb-*specific whole blood IL-31 release assay to define a threshold of positivity for the pleural fluid. Specifically, a concentration ≥23.9 pg/mL was considered positive. Thus, 4 out of the 7 TPE cases which were negative based on detection of IL-31 in the pleural fluid were now positive by whole blood IL-31 release assay. This practice of combining the two tests increased the sensitivity and specificity for the diagnosis of TPE to 94.0% and 95.1%, respectively (Fig. 5 and Table 3).

## Discussion

Dozens of biochemical markers in the pleural fluid are currently being investigated and/or are utilized in the clinic, such as carcinoembryonic antigen (CEA), adenosine deaminase (ADA), interferon-γ (IFN-γ), C-reactive protein (CRP), and lactate dehydrogenase (LDH) for identifying the nature of pleural effusions^2^,^9^,^11^,^13^,^14^,^15^,^16^,^17^,^18^,^19^,^20^,^21^,^22^. For pleural fluid ADA levels >40 U/mL, the sensitivity and specificity for the diagnosis of tuberculous pleurisy were 80.3–100% and 60–100%, respectively^17^. In the study by Porcel and colleagues, of 2104 cases with pleural effusion, the sensitivity and specificity of ADA levels >35 U/mL for the diagnosis of TPE were 93% and 90%, respectively^23^. In addition, numerous studies have shown that the IFN-γ levels were elevated in TPE. A meta-analysis of 22 studies revealed that the mean sensitivity and specificity of this marker to diagnose TPE were 89.0% and 97.0%, respectively^24^. In the differential diagnosis of pleural effusion, the specificity of CEA for the diagnosis of malignant pleural effusion was 100.0%, but the sensitivity was less than 30.0%^25^. Due to the low sensitivity and/or specificity and high variability of the markers mentioned above in the diagnosis of TPE, their clinical applications are limited. In this study, we found that the IL-31 levels in the pleural fluid of TPE patients were significantly higher than those in the pleural fluid of MPE, demonstrating the high diagnostic value of this cytokine.

Interleukin-31 (IL-31) is a recently discovered member of the IL-6 cytokine family. Its receptor heterodimer consists of a unique gp130-like receptor chain, IL-31RA, and the OSM receptor (OSMR)^3^,^4^. IL-31RA is expressed in myeloid monocytes, skin, lung and the epithelial cells, which can lead to the activation of CD4+ and CD8+ T cells^26^,^27^,^28^,^29^,^30^. CD4+ T-cells, particularly activated Th2 cells in the peripheral blood and skin-homing CD45RO CLA+ T-cells have been found to represent a major cellular source of IL-31^31^. However, the biological function of IL-31 is not entirely clear. Preliminary studies have indicated that IL-31 may negatively regulate CD4+ Th2 cytokine secretion^26^,^32^. Moreover, recent studies have examined mouse models and humans and found that IL- 31 participates in itching, alopecia, skin damage, atopic airway hypersensitivity and hepatitis B-related liver failure^8^,^30^,^33^,^34^. Nevertheless, the role of IL-31 in tuberculosis is rarely studied. Thus, we especially enrolled HIV-negative participants in this study and employed *M.tb* specific antigen stimulation to minimize the effect of number of immune cells in the blood on IL-31 detection.

This difference in expression of IL-31 among TPE and MPE groups may be a reflection of their differences in disease pathogenesis. The main mechanism of TPE is a delayed-type hypersensitivity reaction. The consequent inflammatory process increases the pleural vascular permeability to accumulate proteins and specific lymphocytes in the pleural fluid accumulation chamber^35^. During this process, a variety of T cell subsets participate in the tuberculous pleurisy immune response, including Th1, Th2, Th9, Th17, and Th22, which secrete IFN-γ, IL-2, IL-4, IL-6, IL-10, IL-17, IL-22, IL-27 and other cytokines^14^,^21^,^36^,^37^. A previous study found that the number of *M.tb*-specific CD3+ CD4+ T cells in the peripheral blood was much higher in TPE patients than in MPE patients^38^. Furthermore, we analysed the correlation between the IL-31 levels and age in the two groups, but did not identify a correlation in either of the groups. Thus, the combination of the pleural fluid and whole blood assays may improve the diagnostic power of tuberculous pleurisy^38^.

In our study, without antigen stimulation, the plasma IL-31 levels was significantly higher in pneumonia patients with pleural fluid than in the TPE group (*P* = 0.047), but these levels did not differ between the TPE group and the other three groups including MPE, PTB and HC. To avoid the nonspecific secretion of IL-31 in other inflammatory diseases, we employed *M.tb* specific antigen as whole blood stimulator and found that the IL-31 levels in pneumonia patients did not increase by antigen stimulation compared with that without stimulation which could be used for differentiation. Specifically, the antigen-specific IL-31 levels were significantly higher in the TPE group than in the MPE group and the other three groups. Furthermore, the combination of the IL-31 levels in the pleural fluid and the *M.tb* specific whole blood IL-31 release assay was highly specific for the diagnosis of tuberculous pleurisy. The sensitivity and specificity for the diagnosis of TPE were improved to 94.0%, and 95.1%, respectively. Therefore, the IL-31 levels in the pleural fluid and the *M.tb* specific IL-31 in the whole blood constitute a useful biomarker set for the diagnosis of tuberculous pleurisy.

The reason why plasma IL-31 levels were not increased by *M.tb* specific antigen stimulation in PTB group but were so in TPE group (Figs 3 and 4) is unclear. This might be due to secondary suppression of effector T cell response in PTB patients, as a result of a higher antigenic load in PTB than TPE. Consistent with this possibility, our recent study has demonstrated that CD4+ and CD8+ effector T cell responses in the peripheral blood of PTB patients were dramatically decreased compared with latent tuberculosis infection individuals^39^. Future studies that look at Tregs and TGF-β and IL-10 levels in PTB and TPE patients are needed to address the possibility of higher immune suppression as a potential mechanism for the lack of IL-31 production in response to antigen stimulation in PTB patients.

In conclusion, we identified IL-31 to be a novel biomarker for the diagnosis of TPE. The combination of the pleural fluid IL-31 levels with the plasma tuberculosis-specific IL-31 levels constitutes a highly sensitive and specific assay for the differential diagnosis of TPE and MPE. Further studies are needed to validate our findings in larger sample size in clinical settings in the future.

## Materials and Methods

### Participants

A total of 134 participants were enrolled in this study. One hundred and two patients with pleural effusion were consecutively enrolled at Huashan Hospital, Fudan University and Wuxi Infectious Diseases Hospital from January 1, 2011 to May 31, 2013. Fifteen patients with pulmonary tuberculosis were consecutively enrolled at the Wuxi Infectious Diseases Hospital from January 1, 2011 to May 31, 2013. The healthy controls were recruited from volunteers at Fudan University on August 16, 2015.

Tuberculous pleurisy was confirmed by pleural fluid that was culture-positive for *M.tb* and/or a histologically confirmed tuberculosis infection by pleural biopsy. Probable tuberculous pleurisy was diagnosed based on one of the following criteria: *M.tb* culture-positive sputum; *M.tb* culture-positive other biologic specimens; or positive response to anti-tuberculosis medication without other possible causes of pleural effusion^40^.

Patients with pleural effusion who were diagnosed with malignancies were also enrolled in this study as controls. Malignant pleural effusion was diagnosed based on both of the following criteria: (1) *M.tb* culture-negative pleural fluid; (2) a histopathological or cytological diagnosis of pleural tumours.

Pneumonia patients with pleural fluid exhibited symptoms (i.e., fever, cough, sputum, and chest pain), their sputum was culture-negative for *M.tb*, they exhibited exudate pleural effusion, and their acid-fast bacilli smear or culture was negative. Their chest CT images showed substantial lesions with pleural effusion, and the anti-infection treatment was effective^41^. Patients with pulmonary tuberculosis were sputum acid-fast bacillus (AFB) culture-positive and treatment naïve or treated with anti-tuberculosis medication for less than 1 week.

Healthy controls were IGRA-negative and exhibited no evidence of active tuberculosis. All enrolled participants were HIV-negative, had not been diagnosed with cancer, diabetes, autoimmune diseases or other chronic infections (i.e., chronic HBV/HCV infection) and had not received immune-modulating treatments.

This study was approved with written consent by the Ethics Committee of Huashan Hospital, Fudan University with the approval number of 2011–247, and written informed consent was obtained from all the participants. All the methods were carried out in accordance with the approved guidelines and all the experimental protocols were approved by the Human Research Ethics Committee Huashan Hospital, Fudan University.

### Specimen collection

The blood was drawn, and 10 mL of pleural fluid was collected from all the patients by thoracentesis under strict aseptic conditions. The pleural fluid was centrifuged at 300× *g* for 10 min, and the supernatant was frozen at −80 °C for detection.

Three sputum specimens (spot-morning-spot) from each participant were collected, and *M.tb* was detected with an acid-fast bacilli (AFB) smear using the Ziehl-Neelsen method and mycobacterial culture in both Lowenstein Jensen (Biomerieux Inc., I’ Etolie, France) and MGIT tubes (BD BACTC MGIT 960 system).

### IL-31 levels in pleural fluid

Multiplexed bead-based cytokine immunoassays (Bio-Plex cytokine assay kit) were employed to detect the IL-31 levels in the pleural fluid. The detection was conducted according to the instructions provided by manufacturer. The result was measured on a Bio-Plex suspension array system (Bio-Plex 200 System, Bio-Rad Laboratories, Inc.).

### The whole blood IL-31 released assay in response to specific M.tb antigens

The whole blood was stimulated with *M.tb*–specific antigens, including Early Secretory Antigen Target 6 (ESAT-6), Culture Filtrate Protein 10 (CFP-10) and TB7.1. The test consisted of a negative control (whole blood without antigens). Two millilitres of venous blood was collected from each participant and aliquoted into two tubes (*M.tb*-specific antigens and without antigens). The samples were incubated at 37 °C in a humidified 5% CO_2_ incubator for 24 hours. On the second day, the tubes were centrifuged at 3000 rcf for 10 minutes, and the plasma was collected and stored at −80 °C until the IL-31 assay was performed using a Bio-Plex cytokine assay kit and R&D ELISA kit (R&D, USA). The IL-31 levels of the un-stimulated tube was considered background and was subtracted from the results of the antigen-stimulated tube.

### Data analysis

The statistical analysis was performed using GraphPad Prism software (version 5.01; GraphPad Software, Inc.) and MultiExperiment Viewer (version 4.9.0; Dana-Farber Cancer Institute). The data were compared using the non-parametric Mann-Whitney *U*-test. Significance was inferred for *P* < 0.05.

## Additional Information

**How to cite this article**: Gao, Y. *et al*. Potential diagnostic value of serum/pleural fluid IL-31 levels for tuberculous pleural effusion. *Sci. Rep.* **6**, 20607; doi: 10.1038/srep20607 (2016).

## Figures and Tables

**Table 1:** The demographic and clinical characteristics of study participants.

	Total	TPE	MPE	Pneumonia	PTB	HC	*P* value
n	134	50	41	11	15	17	–
Male/Female	93/41	37/13	30/11	9/2	10/5	7/10	0.09
Age, mean (range)	53.2 (12–91)	47.6 (12–84)	64.7 (39–91)	55.8 (29–90)	48.7 (16–78)	38.9 (25–69)	<0.0001
BCG vaccination history, n (%)	83 (61.9)	40 (80.0)	15 (36.6)	6 (54.5)	11 (73.3)	11 (64.7)	<0.0001
Previous TB treatment, n (%)	24 (17.9)	12 (24.0)	0	0	12 (80.0)	0	0.0001
History of exposure to active TB, n (%)	12 (9.0)	6 (12.0)	2 (4.9)	0 (0)	4 (26.7)	0	1.0
Sputum AFB smears or culture positive, n (%)	28 (20.9)	13 (26.0)	0	0 (0)	15 (100)	0	–
*M. tb* detection in pleural effusion							
AFB smears positive, n (%)	2 (1.5)	2 (4.0)	0	–	–	–	–
Culture-positive, n (%)	5 (3.7)	5 (10.0)	0	–	–	–	–
Confirmed TB by pleural biopsy, n (%)	16 (11.9)	16 (32.0)	–	–	–	–	–

TPE: Tuberculous pleural effusion, MPE: Malignant pleural effusion; PTB: Pulmonary tuberculosis; HC: Healthy control; TB: tuberculosis; BCG: bacillus Calmette-Guerin; AFB: acid fast bacilli.

**Table 2:** The diagnostic power of IL-31 of pleural fluid in tuberculous and malignant pleural effusion.

	IL-31 ≥ 67.5 pg/mL	IL-31 < 67.5 pg/mL	Sensitivity	Specificity
TPE (n = 50)	43	7	86.0%	100.0%
MPE (n = 41)	0	41	100.0%	86.0%

TPE: Tuberculous pleural effusion, MPE: Malignant pleural effusion.

**Table 3:** The diagnostic power of the IL-31 levels in the pleural fluid combined with the *M.tb*-specific whole blood IL-31 release assay.

	TPE	MPE	Sensitivity	Specificity
Pleural fluid IL-31 ≥67.5 pg/mL	86.0% (43/50)	0% (0/41)	86.0%	100.0%
Combined with the plasma *M.tb-specific IL-31* ≥23.9 pg/mL	94.0% (47/50)	4.9% (2/41)	94.0%	95.1%

TPE: Tuberculous pleural effusion; MPE: Malignant pleural effusion.

## References

[b1] Porcel J. M. Tuberculous pleural effusion. Lung 187, 263–270 (2009). 19672657 10.1007/s00408-009-9165-3

[b2] Light R. W. Pleural effusions. Med Clin of North Am 95, 1055–1070 (2011). 22032427 10.1016/j.mcna.2011.08.005

[b3] Dillon S. R. . Interleukin 31, a cytokine produced by activated T cells, induces dermatitis in mice. Nat Immunol 5, 752–760 (2004). 15184896 10.1038/ni1084

[b4] Raap U. . Correlation of IL-31 serum levels with severity of atopic dermatitis. J Alergy Clin Immunol 122, 421–423 (2008). 10.1016/j.jaci.2008.05.047 18678344

[b5] Cornelissen C., Luscher-Firzlaff J., Baron J. M. & Luscher B. Signaling by IL-31 and functional consequences. Eur J Cell Bio 91, 552–566 (2012). 21982586 10.1016/j.ejcb.2011.07.006

[b6] Ouyang H., Cheng J., Zheng Y. & Du J. Role of IL-31 in regulation of Th2 cytokine levels in patients with nasal polyps. Eur Arch Otorhinolaryngol 271, 2703–2709 (2014). 24515918 10.1007/s00405-014-2913-x

[b7] Dambacher J. . Interleukin 31 mediates MAP kinase and STAT1/3 activation in intestinal epithelial cells and its expression is upregulated in inflammatory bowel disease. Gut 56, 1257–1265 (2007). 17449633 10.1136/gut.2006.118679 PMC1954980

[b8] Yu X. . The TGF-beta1/IL-31 pathway is up-regulated in patients with acute-on-chronic hepatitis B liver failure and is associated with disease severity and survival. Clinl Vaccine Immunol 22, 484–492 (2015). 10.1128/CVI.00649-14 PMC4412936 25716231

[b9] Light R. W. Update on tuberculous pleural effusion. Respirology 15, 451–458 (2010). 20345583 10.1111/j.1440-1843.2010.01723.x

[b10] Stenger S. & Modlin R. L. Cytotoxic T cell responses to intracellular pathogens. Curr Opin Immuno 10, 471–477 (1998). 10.1016/s0952-7915(98)80123-4 9722925

[b11] Marchi E. . Proinflammatory and antiinflammatory cytokine levels in complicated and noncomplicated parapneumonic pleural effusions. Chest 141, 183–189 (2012). 21680642 10.1378/chest.10-3181

[b12] Flynn J. L. & Ernst J. D. Immune responses in tuberculosis. Curr Opin Immuno 12, 432–436 (2000). 10.1016/s0952-7915(00)00116-3 10899019

[b13] Akarsu S. . The differential diagnostic values of cytokine levels in pleural effusions. Mediators Inflamm 2005, 2–8 (2005). 10.1155/MI.2005.2 PMC1513055 15770060

[b14] Caramori G. . Immune response to Mycobacterium tuberculosis infection in the parietal pleura of patients with tuberculous pleurisy. PloS one 6, e22637 (2011). 21829471 10.1371/journal.pone.0022637 PMC3145659

[b15] Naumnik W., Naumnik B., Niewiarowska K., Ossolinska M. & Chyczewska E. Novel cytokines: IL-27, IL-29, IL-31 and IL-33. Can they be useful in clinical practice at the time diagnosis of lung cancer? Exp Oncol 34, 348–353 (2012). 23302994

[b16] Nepal A. K. . Adenosine deaminase in CSF and pleural fluid for diagnosis of tubercular meningitis and pulmonary tuberculosis. Nepal Med Coll J 14, 275–277 (2012). 24579533

[b17] da Silva C. T. Jr., Behrsin R. F., Cardoso G. P. & de Araujo E. G. Evaluation of adenosine deaminase activity for the diagnosis of pleural TB in lymphocytic pleural effusions. Biomark Med 7, 113–118 (2013). 23387492 10.2217/bmm.12.89

[b18] Keng L. T. . Evaluating pleural ADA, ADA2, IFN-gamma and IGRA for diagnosing tuberculous pleurisy. J Infect 67, 294–302 (2013). 23796864 10.1016/j.jinf.2013.05.009

[b19] Porcel J. M. & Light R. W. Pleural effusions. Dis Mont 59, 29–57 (2013). 10.1016/j.disamonth.2012.11.002 23374395

[b20] Islam A. . Role of adenosine deaminase in diagnosis of tubercular pleural effusion. Mymensingh Med J 23, 24–27 (2014). 24584368

[b21] Shu C. C. . Diagnostic role of inflammatory and anti-inflammatory cytokines and effector molecules of cytotoxic T lymphocytes in tuberculous pleural effusion. Respirology 20, 147–154 (2015). 25355638 10.1111/resp.12414

[b22] Porcel J. M. . Biomarkers of infection for the differential diagnosis of pleural effusions. Eur Respir J 34, 1383–1389 (2009). 19541708 10.1183/09031936.00197208

[b23] Porcel J. M., Esquerda A. & Bielsa S. Diagnostic performance of adenosine deaminase activity in pleural fluid: a single-center experience with over 2100 consecutive patients. Eur J Intern Med 21, 419–423 (2010). 20816597 10.1016/j.ejim.2010.03.011

[b24] Jiang J., Shi H. Z., Liang Q. L., Qin S. M. & Qin X. J. Diagnostic value of interferon-gamma in tuberculous pleurisy: a metaanalysis. Chest 131, 1133–1141 (2007). 17426220 10.1378/chest.06-2273

[b25] Porcel J. M. . Use of a panel of tumor markers (carcinoembryonic antigen, cancer antigen 125, carbohydrate antigen 15-3, and cytokeratin 19 fragments) in pleural fluid for the differential diagnosis of benign and malignant effusions. Chest 126, 1757–1763 (2004). 15596670 10.1378/chest.126.6.1757

[b26] Perrigoue J. G. . IL-31-IL-31R interactions negatively regulate type 2 inflammation in the lung. J Exp Med 204, 481–487 (2007). 17353366 10.1084/jem.20061791 PMC2137900

[b27] Jawa R. S., . Regulated expression of the IL-31 receptor in bronchial and alveolar epithelial cells, pulmonary fibroblasts, and pulmonary macrophages. J Interferon Cytokine Res 28, 207–219 (2008). 18439099 10.1089/jir.2007.0057

[b28] Perrigoue J. G., Zaph C., Guild K., Du Y. & Artis D. IL-31-IL-31R interactions limit the magnitude of Th2 cytokine-dependent immunity and inflammation following intestinal helminth infection. J Immunol 182, 6088–6094 (2009). 19414760 10.4049/jimmunol.0802459 PMC2828776

[b29] Kasraie S., Niebuhr M. & Werfel T. Interleukin (IL)-31 induces pro-inflammatory cytokines in human monocytes and macrophages following stimulation with staphylococcal exotoxins. Allergy 65, 712–721 (2010). 19889120 10.1111/j.1398-9995.2009.02255.x

[b30] Raap U. . IL-31 significantly correlates with disease activity and Th2 cytokine levels in children with atopic dermatitis. Pediatr Allergy Immunol 23, 285–288 (2012). 22509760 10.1111/j.1399-3038.2011.01241.x

[b31] Rabenhorst A. & Hartmann K. Interleukin-31: a novel diagnostic marker of allergic diseases. Curr Allergy Asthma Rep 14, 423 (2014) 24510535 10.1007/s11882-014-0423-y

[b32] Szegedi K. . Increased frequencies of IL-31-producing T cells are found in chronic atopic dermatitis skin. Exp Dermatol 21, 431–436 (2012). 22621183 10.1111/j.1600-0625.2012.01487.x

[b33] Arai I., Tsuji M., Takeda H., Akiyama N. & Saito S. A single dose of interleukin-31 (IL-31) causes continuous itch-associated scratching behaviour in mice. Exp Dermatol 22, 669–671 (2013). 24079740 10.1111/exd.12222

[b34] Arai I. . Repeated administration of IL-31 upregulates IL-31 receptor A (IL-31RA) in dorsal root ganglia and causes severe itch-associated scratching behaviour in mice. Exp Dermatol 24, 75–78 (2015). 25381841 10.1111/exd.12587

[b35] Jeon D. Tuberculous pleurisy: an update. Tuber Resp Diss (Seoul) 76, 153–159 (2014). 10.4046/trd.2014.76.4.153 PMC4021261 24851127

[b36] Ye Z. J. . Differentiation and recruitment of IL-22-producing helper T cells stimulated by pleural mesothelial cells in tuberculous pleurisy. Am J Respir Crit Care Med 185, 660–669 (2012). 22199006 10.1164/rccm.201107-1198OC

[b37] Yang W. B. . Cell origins and diagnostic accuracy of interleukin 27 in pleural effusions. PloS One 7, e40450 (2012). 22792330 10.1371/journal.pone.0040450 PMC3392223

[b38] Prabha C., Jalapathy K. V., Matsa R. P. & Das S. D. Differential T helper cell response in tuberculous pleuritis. Indian J Med Microbiol 25, 18–23 (2007). 17377347 10.4103/0255-0857.31056

[b39] Gao Y. . Characterization of CD4/CD8+ alphabeta and Vgamma2Vdelta2+ T cells in HIV-negative individuals with different Mycobacterium tuberculosis infection statuses. Hum Immunol 76, 801–807 (2015). 26429305 10.1016/j.humimm.2015.09.039

[b40] Moon J. W. . The clinical utility of polymerase chain reaction for the diagnosis of pleural tuberculosis. Clin Infect Dis 41, 660–666 (2005). 16080088 10.1086/432474

[b41] Eccles S., Pincus C., Higgins B. & Woodhead M. Diagnosis and management of community and hospital acquired pneumonia in adults: summary of NICE guidance. BMJ 349, g6722 (2014). 25471702 10.1136/bmj.g6722

